# Logistic regression model can reduce unnecessary artificial liver support in hepatitis B virus-associated acute-on-chronic liver failure: decision curve analysis

**DOI:** 10.1186/s12911-016-0302-7

**Published:** 2016-06-04

**Authors:** Gang Qin, Zhao-Lian Bian, Yi Shen, Lei Zhang, Xiao-Hong Zhu, Yan-Mei Liu, Jian-Guo Shao

**Affiliations:** Center for Liver Diseases, Nantong Third People’s Hospital, Nantong University, Jiangsu, China; Department of Epidemiology and Medical Statistics, School of Public Health, Nantong University, Jiangsu, China; Faculty of Medicine, Nursing and Health Science, Monash University, Melbourne, VIC Australia

**Keywords:** Model for end-stage liver disease, Logistic regression model, Acute-on-chronic liver failure, Hepatitis B virus, Decision curve analysis

## Abstract

**Background:**

Several models have been proposed to predict the short-term outcome of acute-on-chronic liver failure (ACLF) after treatment. We aimed to determine whether better decisions for artificial liver support system (ALSS) treatment could be made with a model than without, through decision curve analysis (DCA).

**Methods:**

The medical profiles of a cohort of 232 patients with hepatitis B virus (HBV)-associated ACLF were retrospectively analyzed to explore the role of plasma prothrombin activity (PTA), model for end-stage liver disease (MELD) and logistic regression model (LRM) in identifying patients who could benefit from ALSS. The accuracy and reliability of PTA, MELD and LRM were evaluated with previously reported cutoffs. DCA was performed to evaluate the clinical role of these models in predicting the treatment outcome.

**Results:**

With the cut-off value of 0.2, LRM had sensitivity of 92.6 %, specificity of 42.3 % and an area under the receiving operating characteristic curve (AUC) of 0.68, which showed superior discrimination over PTA and MELD. DCA revealed that the LRM-guided ALSS treatment was superior over other strategies including “treating all” and MELD-guided therapy, for the midrange threshold probabilities of 16 to 64 %.

**Conclusions:**

The use of LRM-guided ALSS treatment could increase both the accuracy and efficiency of this procedure, allowing the avoidance of unnecessary ALSS.

## Background

Acute-on-chronic liver failure (ACLF) is characterized by severe jaundice, coagulopathy, hepatic encephalopathy (HE), and high morbidity and mortality. Hepatitis B virus (HBV) infection is the commonest cause of ACLF in the Asian region [1]. Currently, the treatment of HBV-associated ACLF (HBV-ACLF) is based on two main regimes – antiviral treatment and artificial liver support system (ALSS) in China [2]. Antiviral therapy with nucleos(t)ide analogues has been proven to significantly improve prognosis and survival rate in HBV-ACLF patients [3]. However, the use of the ALSS has shown a variable range of safety and efficiency in several clinical trials [4–8]. In the last decade, several models have been proposed to predict the short-term outcome of HBV-ACLF after treatment. For instance, antiviral treatment can significantly decrease the 3-month mortality only in patients with the model for end-stage liver disease (MELD) score below 30, compared with those with higher MELD scores [9]. Another study suggested that ACLF patients with lower MELD scores showed significantly improved prognosis compared with those with higher MELD scores [10]. Therefore, the variable results of the ALSS treatment in terms of cost-effectiveness might be explained by the lack of guidance of such predictive models. An ideal prediction model may provide objective information about whether future treatment is likely to result in a favorable outcome or survival.

Recently, some studies revealed higher diagnostic accuracy for predictive models which combined liver dysfunction with etiological factor (e.g. HBV). In particular, Zheng et al., in a population of 452 patients with diagnosis of HBV-ACLF, established the logistic regression model (LRM) score, with an area under the receiving operating characteristic curve (AUC) of 0.844. With the cutoff of 0.2, a sensitivity of 86.7 and specificity of 75.5 % were reported [11]. LRM has shown promising results for prognosis prediction in HBV-ACLF ever since its introduction into clinical application. Yang et al. compared the predictive performance of MELD with that of LRM in a population of 273 HBV-ACLF patients. In ACLF patients with liver cirrhosis (LC), the AUC of LRM (0.851) was comparable with that of MELD (0.840). Yet, in patients with non-cirrhotic ACLF, the AUC of LRM (0.897) was significantly higher than that of MELD (0.758) [12].

In a previous study, we reported that ALSS could improve short- and long-term prognosis in patients with HBV-ACLF [13]. Here we reanalyzed the data in order to determine whether better decisions for ALSS treatment could be made with a model (e.g. MELD or LRM) than without, through decision curve analysis.

## Methods

### Study patients

From January 2003 to December 2007, all patients admitted to our hospital with the diagnosis of HBV-ACLF according to the Chinese guidelines [14, 15] were screened. Eligible patients were enrolled with the following criteria: (i) aged between 18 and 70 years; (ii) presumptively diagnosed as hepatitis B surface antigen (HBsAg) carrier, chronic hepatitis B (CHB) or HBV-related liver cirrhosis (HBC); (iii) progressive hyperbilirubinemia, with serum total bilirubin (TBil) ≥10 mg/dL; (iv) coagulopathy with plasma prothrombin activity (PTA) ≤40 % or international normalized ratio (INR) >1.5; (v) within 4 weeks from symptom onset complicated by ascites and/or HE. ACLF is further classified into early stage (30 % < PTA ≤ 40 %) and middle-to-end stage (PTA ≤ 30 %) [15]. Some patients were excluded according to the following criteria: acute HBV infection, super-infection with other viruses, chronic liver failure, coexistence of hepatocellular carcinoma (HCC), severe gastrointestinal bleeding, pregnancy, or liver transplant recipients.

The primary endpoint was 3-month survival. The secondary endpoint was survival at 5 years after diagnosis of ACLF.

The medical profiles of the patients were retrospectively analyzed in the present study. Our study protocol was approved by the institutional review board (IRB) of Nantong Third People’s Hospital, Nantong University and conducted in accord with the ethical guidelines of the Declaration of Helsinki 1975. Patient consents were waived by the same IRB.

### MELD calculation

MELD score (range from 6 to 40) was calculated according to the standard formula [16]. MELD = 11.2 × ln(INR) + 9.6 × ln[creatinine (mg/dL)] + 3.8 × ln[TBil (mg/dL)] + 6.4 (constant for liver disease etiology). Laboratory values of bilirubin, INR or creatinine less than 1 were rounded off to 1, in order to avoid negative scores. Creatinine greater than 4 mg/dL or with renal replacement therapy was capped at 4 mg/dL. In addition, the factor for etiology of liver disease was not used.

### LRM calculation

LRM score was calculated in accordance with the original reference [11]. LRM =−1.343 + 0.772 × HE + 2.279 × HRS + 0.85 × LC + 1.026 × HBeAg − 2.117 × PTA/age. For HE, hepatorenal syndrome (HRS), LC and hepatitis B e antigen (HBeAg), yes/positive = 1 while no/negative = 0. The score was rounded to the nearest tenth.

### ALSS treatment

The patients with HBV-ACLF were assigned randomly to groups either given ALSS combined with standard medical therapy (SMT) or only SMT. The randomization was conducted by the Department of Epidemiology and Medical Statistics, Nantong University based on the SAS module, as we described in a previous study [13].

### Statistical analysis

The accuracy of PTA, MELD or LRM was evaluated separately. For each model, the AUC, sensitivity, specificity, positive predictive value (PPV), negative predictive value (NPV), as well as diagnostic odds ratio (DOR), with the 95 % confidence intervals (CIs) were evaluated.

**Fig. 1:**
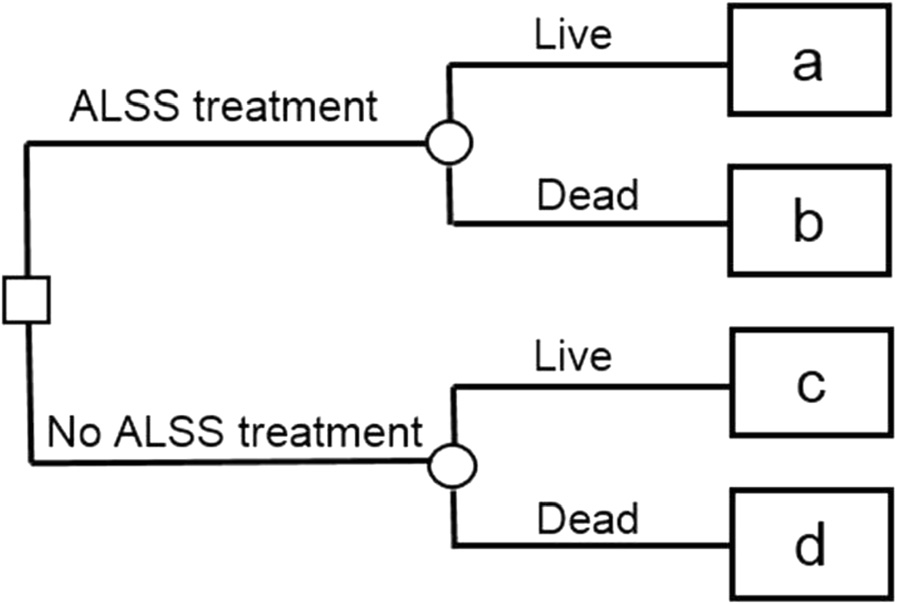
A decision tree of ALSS treatment for HBV-ACLF patients. The a, b, c, and d give, respectively, the value of true positive, false positive, false negative, and true negative

A simple decision tree is constructed with the HBV-ACLF patients in Fig. 1. The splitting parameter is recommendation for ALSS treatment or not. The a, b, c and d showing live or dead cases in each group, give the values of true positive, false positive, false negative, and true negative respectively. A cost-effective analysis, proposed by Vickers [17–19], was performed for each model using decision curve analysis (DCA). The net benefit of a model was estimated by the difference between the number of true-positive and false-positive, weighted by the odds of the specific threshold probability (Pt). A threshold value of, for example, 20 % indicates that survival probability of a treated patient is 80:20 = 4 times higher than the complications of an unnecessarily treated patient. Furthermore, using LRM for a particular patient, Pt of 10 % indicates that his possibility of LRM < 0.2 (ALSS treatment recommended) is 90:10 = 9 times higher than that of LRM ≥ 0.2 (ALSS treatment unnecessary). The net benefit of a model compared with another model or with the reference net benefit could be interpreted as the net increase in the proportion of cases identified. We calculate net benefit using the following formula [17]: net benefit = a/N – b/N × [Pt/(1-Pt)]. We set a zero net benefit by assuming no patient undergoing ALSS; on the contrary, we calculated the reference by assuming that all patients had undergone ALSS treatment. For any given threshold probability (Pt) cut point, the preferred model would be that with greater net benefit. The reduction in the number of unnecessary ALSS is calculated using the following formula [17]: reduction of avoidable ALSS treatment per 100 patients = (net benefit of the model – net benefit of treat all)/[Pt/(1 – Pt)] × 100. The value is net of false negatives and is hence the equivalent to the reduction number of unnecessary ALSS without a decrease in the number of patients with ACLF who duly need ALSS.

All statistical analysis, including the DCA and plots implementation, were done using Stata version 13 (StataCorp, TX, USA). DCA analysis was performed using the code found at  https://www.mskcc.org/departments/epidemiologybiostatistics/health-outcomes/decision-curve-analysis-01 according to its tutorials.

## Results

### Patient characteristics

A total of 283 patients diagnosed with HBV-ACLF were screened for inclusion in this study cohort. Among these, 51 patients were excluded from this study: five for age over 70 years, eight for super-infection with hepatitis E virus, two for coexistence of HCC, seven for liver transplantation, 29 for incomplete data. Thus the final cohort compromised 232 patients with complete medical records.

**Fig. 2:**
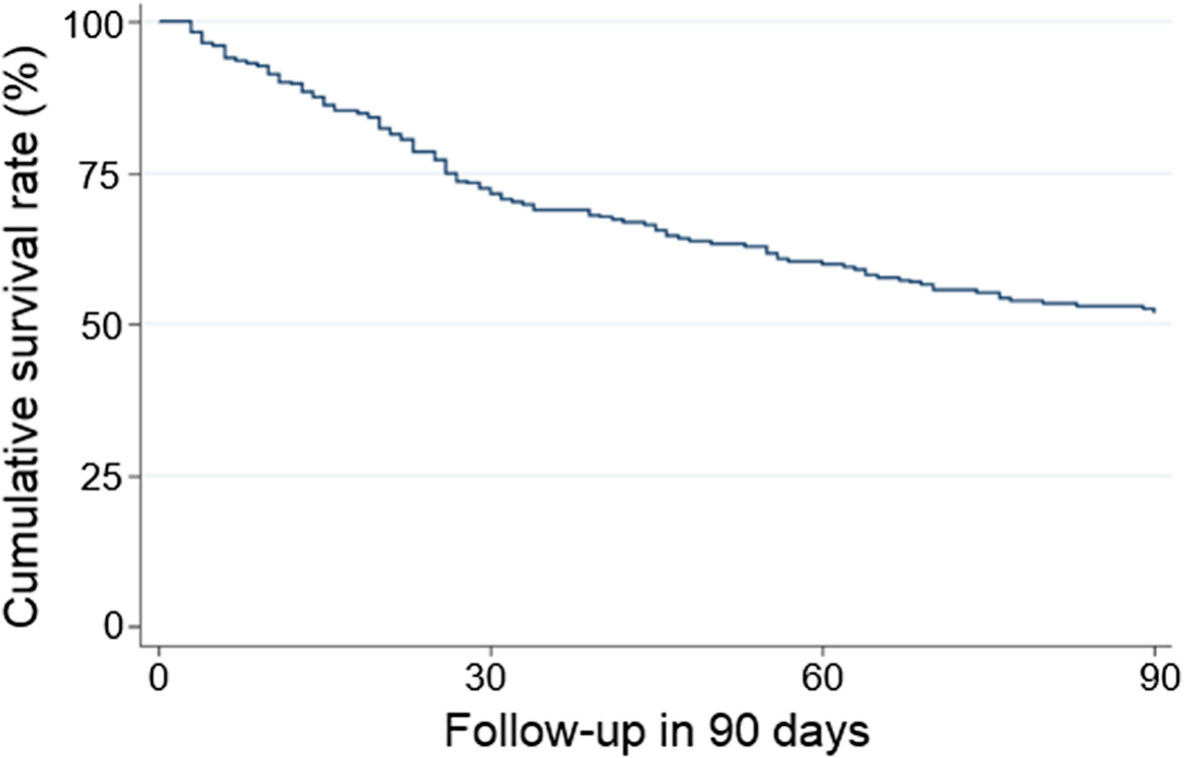
Cumulative survival in HBV-ACLF patients over follow-up of 90 days

The study cohort had a median (range) age of 45 (21–69) years, with 77 % male (Table 1). Cirrhosis was preexisted in 112 (48.3 %) patients. The mean HBV DNA was 4.1 ± 2.5 lg copies/ml and 142 patients (61.2 %) were hepatitis B e antigen (HBeAg) positive. Moreover, the mean (SD) value of serum bilirubin was 22.2 (9.2) mg/dL, PTA 27.1 % (16.8 %), INR 4.2 (2.2), serum creatinine 0.93 (0.74) mg/dL, and albumin 32.1 (5.0) g/L. The most common complications were ascites (194 patients; 83.6 %), spontaneous bacterial peritonitis (SBP, 152 patients, 65.5 %), HE (64 patients; 27.67 %), and HRS (37 patients; 16 %). Of these, 104 (44.8 %) patients received ALSS therapy. 121 (52.2 %) patients survived the first 3 months of follow-up (Table 1, Fig. 2).

**Table 1:** Demographic, clinical and laboratory features of the study patients

Characteristic	Value
Number of patients	232
Male/female	178 (76.7 %)/54 (23.3 %)
Age (years)	46.1 ± 10.5 (45; 21–69)
HBeAg positivity	142 (61.2 %)
HBV DNA (lg copies/mL)	4.1 ± 2.5
TBil (mg/dL)	22.2 ± 9.2
Cr (mg/dL)	0.93 ± 0.74
PTA (%)	27.1 ± 16.8
INR	4.2 ± 2.2
Albumin (g/L)	32.1 ± 5.0
Preexisting cirrhosis	112 (48.3 %)
Ascites	194 (83.6 %)
SBP	152 (65.5 %)
HE	64 (27.6 %)
HRS	37 (16.0 %)
MELD	29.0 ± 5.4
LRM	−0.6 ± 1.4
ALSS treatment	104 (44.8 %)
3-month survival	121(52.2 %)

*Note*: Data presented as mean ± standard deviation or n (%)

*TBil* total bilirubin, *PTA* prothrombin activity, *INR* international normalized ratio, *SBP* spontaneous bacterial peritonitis, *HE* hepatic encephalopathy, *HRS* hepatorenal, syndrome, *MELD* model for end-stage liver disease, *LRM* logistic regression model, *ALSS* artificial liver support system

### Predictive performance of the models

As recommended by previous studies, the cutoffs for PTA, MELD and LRM models were set for 30 %, 30 and 0.2 respectively. With the cut-off value of 30 %, PTA had sensitivity of 39.7 %, specificity of 77.5 %, PPV of 65.8 %, NPV of 54.4 %, AUC of 0.59 and DOR of 2.26. With the cut-off value of 30, MELD had sensitivity of 55 %, specificity of 70.2 %, PPV of 62.9 %, NPV of 63 %, AUC of 0.63 and DOR of 2.88. With the cut-off value of 0.2, LRM had sensitivity of 92.6 %, specificity of 42.3 %, PPV of 63.6 %, NPV of 83.9 %, AUC of 0.68 and DOR of 9.14. LRM showed superior discrimination over the others (Table 2).

**Table 2:** Performance the models to predict 3-month outcome with the recommended cutoffs

Model	PTA	MELD	LRM
cutoff	30 %	30	0.2
Sensitivity	39.7 %	55 %	92.6 %
Specificity	77.5 %	70.2 %	42.3 %
PPV	65.8 %	62.9 %	63.6 %
NPV	51.4 %	63 %	83.9 %
AUC	0.59 (0.53–0.64)	0.63 (0.56–0.69)	0.68 (0.62–0.73)
DOR	2.26 (1.28–4.01)	2.88 (1.68–4.93)	9.14 (4.25–19.6)

*PTA* prothrombin activity, *MELD* model for end-stage liver disease, *LRM* logistic, regression model, *PPV* positive predictive value, *NPV* negative predictive value, *AUC* area under the receiving operating characteristic curve, *DOR* diagnostic odds ratio

### Decision curve analysis

In Table 3, where LRM model was applied, the total number of patients (N) is 232, the true-positive count is 112, and the false-positive count is 64. The net benefit for LRM-guided treatment is therefore (112/232) – (64/232) × [Pt/(1-Pt)]. The true- and false-positive counts for the “treat all” strategy simply are the number of patients with and without ALSS treatment respectively [17]. Calculating net benefit for “treat all” gives (121/232) – (111/232) × [Pt/(1-Pt)] while removing LRM model in all patients.

**Table 3:** Relationship between True ALSS Treatment and Result of a LRM-guided ALSS Treatment

	ALSS		
	*N* = 232	Live	Dead
LRM model:	Yes	112	64
LRM < 0.2	No	9	47

*Note*: net benefit of LRM model = 112/232 – 64/232× [Pt/(1-Pt)], net benefit of treat all = 121/232 – (111/233) × [Pt/(1-Pt)]

*ALSS* artificial liver support system, *LRM* logistic regression model, *Pt* threshold probability

**Fig. 3:**
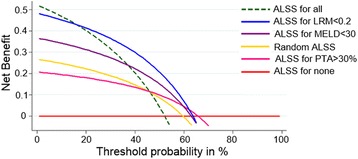
Decision curve for prediction of net benefit in ALSS treatment for HBV-ACLF patients. *Red line*: assume no patient was treated with ALSS (“treat none”). *Green dash line*: assume all patients were treated with ALSS (“treat all). *Pink line*: assume only patients with higher PTA (>30 %) were treated with ALSS. *Yellow line*: patients were treated with random ALSS assignment. *Purple line*: assume only patients with low MELD scores (<30) were treated with ALSS. Blue line: assume only patients with low LRM scores (<0.2) were treated with ALSS

Decision curves for ACLF treatment, with the 3 analyzed models, were plotted in Fig. 3 to estimate the results in a clinical context. The net benefit of the single marker PTA was similar to the net benefit of random ALSS assignment. LRM-guided ALSS compared with “treating all” strategy lead to a greater net benefit for ever probability threshold starting from 16 %. Besides, it was always higher than the net benefit of MELD-guided strategy. Both the LRM and MELD model showed a superior net benefit compared with the random ALSS assignment. The net benefit of 0 at a Pt of 16 % could be interpreted in terms that if we perform ALSS based on LRM score, the consequence is equivalent to ALSS for all patients. Furthermore, at a Pt of 64 %, the net benefit for the prediction model is 0.321 greater than assuming all patients need ALSS. The net benefit formula was used to calculate which was the equivalent of a net 0.321 × 100/(0.64/0.36) = 18 fewer false positive results per 100 patients. It means that use of the prediction model might result in the equivalent of 18 % fewer ALSS in patients with ACLF, with no increase in the number of patients who need ALSS left untreated. For higher Pt (>64 %), the option to “not treat” is preferred and neither predictive model has value (Fig. 3, Table 4).

**Table 4:** Net Benefit for ALSS for All ACLF Patients or According to LRM, Using a Threshold of Pt

Pt (%)	Net Benefit		Advantage of LRM-guided ALSS	
	ALSS for All	LRM-guided ALSS	Net Benefit	Reduction in Avoidable ALSS per 100 Patients
16	0.430	0.430	0	0
20	0.402	0.414	0.012	5
30	0.317	0.365	0.048	11
40	0.203	0.299	0.096	14
50	0.043	0.207	0.164	16
60	−0.197	0.069	0.266	18
64	−0.329	−0.008	0.321	18

*Note*: The reduction in the number of unnecessary ALSS per 100 patients is calculated as follows: (net benefit of the model – net benefit of treat all)/[Pt/(1 – Pt)] × 100

*ALSS* artificial liver support system, *ACLF*, acute-on-chronic liver failure, *LRM* logistic regression model, *Pt* threshold probability of risk

## Discussion

Artificial liver support system was first applied to treat acute liver failure in 1970s with the attempt to replace certain detoxification function of the liver. On the one hand, the therapy cost is as expensive as nearly $US2500 for each session of ALSS in China. The incidence of adverse events (i.e. bleeding, hypotension, infection, coagulopathy, and catheter-related events) were reported [5, 6, 8, 13, 20]. On the other hand, several clinical trials and systemic reviews suggested that ALSS could reduce mortality in ACLF patients compared with standard medical therapy [21–24]. Therefore, ALSS has been recommended as one important method for the treatment of ACLF [1, 15, 25, 26].

It has been extensively debated whether to treat all ACLF patients or to treat selected patients. Some efforts have been made toward the identification of factors or models for predicting the prognosis after ALSS. For instance, many factors, including HE, PTA, bilirubin, creatinine, sodium, preexisting cirrhosis and age, were found as independent predictors for the short-term survival rate in ACLF [13]. Several models such as MELD and LRM, with recommended cutoff values, have been proposed to predict the survival outcomes of ACLF patients [11, 27]. Since their introduction into clinical practice, the MELD and LRM scores have been tested in quite a few studies. Yet wide range of sensitivity and specificity has been reported in predicting mortality of ACLF patients [9, 10, 12, 28]. Despite the well-known utility of MELD in allocating donor livers [27], the clinical utility of MELD and other models for other treatments remains unclear.

Although discrimination and calibration are essential aspects of a prediction model, they do not evaluate clinical usefulness such as the ability to make better decisions with a model than without. Decision curve analysis is a method for assessing the benefits of a model through a range of patient preferences in accepting risk of overtreatment and undertreatment to facilitate decision making [29, 30]. The hypothesis in our study was that we may make better decisions for ALSS treatment with a model (e.g. MELD or LRM) than without. For a prediction model aiming to guide therapeutic decisions, a cut-off is required for the decision threshold. Right at the threshold, the likelihood of benefit, e.g. improved survival as a result of ALSS treatment, exactly balances the likelihood of harm, e.g. adverse events and expensive costs. However, as empirical evidence for the relative weight of benefits and harms is often lacking, it is always not easy to define a threshold.

In this study, we applied the DCA to evaluate the cost/benefit ratio of one single marker (i.e. PTA) and two models (i.e. MELD and LRM). The utility of PTA alone resulted in no more net benefit gain than random ALSS assignment. Using the MELD or LRM scores, some number of unnecessary ALSS treatment could be avoided at the cost that only a small proportion of patients with HBV-ACLF being advised not to undergo ALSS treatment. Starting from the cutoff of 16 %, the net benefit gain of LRM-guided strategy starts to be remarkable. The DCA results showed that for patients with threshold probabilities between 0 and 16 %, relatively preferring for empirical therapy, the net benefit is the greatest if all patients are treated. Across this range of threshold probabilities, patients tend to be more concerned about missing a timely treatment than about receiving unnecessary one. For the midrange threshold probabilities between 16 and 64 %, the LRM-guided ALSS therapy is superior to other strategies, including the MELD score. For higher thresholds (>64 %) at which patients appear be more concerned about unnecessary treatment than missed one, the option to not treat is preferred and none of the predictive models has value.

Admittedly, there are some limitations in our study. First, we applied the decision curve analysis theory retrospectively with our cohort. Second, our findings were based on a small sample size. Finally, the present methodology may be appropriate for point decision making but not necessarily for decisions which reoccur over time, because the scores may frequently change in the natural history of ACLF.

## Conclusions

Our findings indicate that the use of LRM-guided ALSS treatment could increase both the accuracy and efficiency of this procedure. Promising results from studies on the novel LRM score for ACLF prognosis may lead to better accuracy when predicting post-treatment outcomes in the near future, allowing the avoidance of unnecessary ALSS.

## Abbreviations

ACLF, acute-on-chronic liver failure; ALSS, artificial liver support system; AUC, area under the receiving operating characteristic curve; CHB, chronic hepatitis B; CI, confidence interval; DCA, decision curve analysis; DOR, diagnostic odds ratio; HBC, HBV-related cirrhosis; HBeAg, hepatitis B e antigen; HBsAg, hepatitis B surface antigen; HBV, hepatitis B virus; HCC, hepatocellular carcinoma; HE, hepatic encephalopathy; HRS, hepatorenal syndrome; INR, international normalized ratio; IRB, institutional review board; LC, liver cirrhosis; LRM, logistic regression model; MELD, model for end-stage liver disease; NPV, negative predictive value; PPV, positive predictive value; Pt, threshold probability; PTA, prothrombin activity; SBP, spontaneous bacterial peritonitis; SMT, standard medical therapy; TBil, total bilirubin
